# Polyetherimide Foams Filled with Low Content of Graphene Nanoplatelets Prepared by scCO_2_ Dissolution

**DOI:** 10.3390/polym11020328

**Published:** 2019-02-13

**Authors:** Hooman Abbasi, Marcelo Antunes, José Ignacio Velasco

**Affiliations:** Centre Català del Plàstic, Departament de Ciència dels Materials i Enginyeria Metal lúrgica, Universitat Politècnica de Catalunya (UPC Barcelona Tech), C/Colom 114, E-08222 Terrassa, Barcelona, Spain; hooman.abbasi@upc.edu (H.A.); jose.ignacio.velasco@upc.edu (J.I.V.)

**Keywords:** polyetherimide foams, graphene, multifunctional foams, ultrasonication, scCO_2_, electrical conductivity

## Abstract

Polyetherimide (PEI) foams with graphene nanoplatelets (GnP) were prepared by supercritical carbon dioxide (scCO_2_) dissolution. Foam precursors were prepared by melt-mixing PEI with variable amounts of ultrasonicated GnP (0.1–2.0 wt %) and foamed by one-step scCO_2_ foaming. While the addition of GnP did not significantly modify the cellular structure of the foams, melt-mixing and foaming induced a better dispersion of GnP throughout the foams. There were minor changes in the degradation behaviour of the foams with adding GnP. Although the residue resulting from burning increased with augmenting the amount of GnP, foams showed a slight acceleration in their primary stages of degradation with increasing GnP content. A clear increasing trend was observed for the normalized storage modulus of the foams with incrementing density. The electrical conductivity of the foams significantly improved by approximately six orders of magnitude with only adding 1.5 wt % of GnP, related to an improved dispersion of GnP through a combination of ultrasonication, melt-mixing and one-step foaming, leading to the formation of a more effective GnP conductive network. As a result of their final combined properties, PEI-GnP foams could find use in applications such as electrostatic discharge (ESD) or electromagnetic interference (EMI) shielding.

## 1. Introduction

Polyetherimide (PEI) is a high-performance thermoplastic that has proven to be a viable candidate in advanced applications in cutting edge sectors, such as aerospace, due to its outstanding properties, including, but not limited to, high mechanical performance, high chemical and inherently high flame resistance, thermal and dimensional stability, low smoke generation, and transparency [1]. Weight reduction by means of foaming has been proven as one of the most promising strategies for cost reduction and for attaining functional characteristics for applications such as EMI shielding [2]. The properties of PEI-based nanocomposite foams prepared using water vapour-induced phase separation (WVIPS) have been investigated in depth and the effect of carbon-based nanoparticles on the physical properties of these foams has been studied, showing promising results in terms of simultaneously enhancing the mechanical properties and electrical conductivity [3,4,5,6,7].

Another foaming technique with characteristics closer to that of industrial foaming processes involves the dissolution of a gas in a polymer precursor in a semisolid-state, i.e., below its melting temperature (semicrystalline polymers) or below its glass transition temperature (amorphous polymers) and subsequent foaming by either applying a sudden drop of pressure (called one-step or solid-state batch foaming) or heating the gas-saturated precursor above its glass transition temperature after a slow decompression. Both methods have been used to prepare various foams with homogeneous microcellular structures using polymers such as acrylonitrile–butadiene–styrene (ABS) [8], polymethylmethacrylate (PMMA) [9], poly(styrene-co-acrylonitrile) (SAN)/chlorinated polyethylene (CPE) blend [10], polycarbonate (PC) [11] or polyethylene terephthalate (PET) [12]. PEI-based foams have also been prepared in this way using sub–critical CO_2_ as the blowing agent [13,14]. 

Carbon-based nanoparticles (carbon nanotubes, CNT; graphene and graphene-based materials; and carbon nanofibres, CNF) have recently received significant attention due to their outstanding combination of mechanical, thermal, and electrical properties [15,16]. Particularly, graphene and graphene-based materials, such as graphene oxide, reduced graphene oxide, or graphene nanoplatelets, offer great possibilities in terms of improving multiple aspects of polymers due to their high aspect ratio and exceptional mechanical, thermal and electrical characteristics [17,18,19]. For instance, the addition of GnP/CNT hybrids to PEI-based foams prepared using WVIPS led to significant improvements of their electrical conductivity, reaching values as high as 8.8 × 10^−3^ S/m for 1 wt % of each filler [5]. Our previous study showed that by achieving a proper GnP dispersion through ultrasonication, the electrical conductivity value of PEI-based nanocomposites foamed via WVIPS could reach as high as 1.7 × 10^−1^ S/m for foams containing 10 wt % GnP.

Although the preparation and characterization of PEI foams using sub–critical and supercritical CO_2_ (scCO_2_) have been carried out [13,14,20], not many studies have considered the investigation of PEI-based nanocomposite foams. Carbon-based nanoparticles in particular, have presented promising results in the creation of multifunctional foams. The combination of high-performance polymers with these functional nanoparticles could result in outstanding nanocomposite foams with enhanced specific properties. Additionally, scCO_2_ foaming has shown promising results in improving the dispersion level of nanoparticles throughout the polymer matrix after foaming. Recent studies on PC-based foams containing GnP have shown that foaming could improve their electrical conductivity and EMI shielding effectiveness by inducing a better exfoliation of graphene stacks and reducing the effective inter-particle distance [21]. Another study on PC-based foams [22] suggests that the electrical conductivity of foams prepared by scCO_2_ dissolution could be enhanced and surpass their respective unfoamed nanocomposites due to improved homogenous dispersion of GnP after foaming.

Furthermore, studies have suggested that the addition of nano-sized particles, such as carbon nanotubes and graphene, to foams prepared by CO_2_ dissolution could improve cellular structure homogeneity, increase cell density, reduce cell size and, at the same time, reinforce the matrix [23,24].

This article considers investigating the effects of foaming by scCO_2_ on the cellular structure, thermal, mechanical, and electrical properties of PEI foams containing variable concentrations of GnP (0.1–2.0 wt %), with the objective of developing novel lightweight materials for advanced applications, such as EMI shielding, ESD, and fuel cells.

## 2. Materials and Methods 

Polyetherimide (PEI), commercially known as Ultem 1000, was purchased from Sabic (Riyadh, Saudi Arabia). PEI Ultem 1000 is a thermoplastic with a density of 1.27 g/cm^3^ and a glass transition temperature (*T*_g_) of 217 °C. Graphene nanoplatelets (GnP), with the commercial name of xGnP M-15, were supplied by XG Sciences (Lansing, MI, USA). These nanoparticles have a density of 2.2 g/cm^3^ and are formed by stacks of individual graphene nanoplatelets. These stacks have an average thickness of 6–8 nm, a lateral size of 15 µm and a surface area of 120–150 m^2^/g. As reported by the manufacturer, the electrical conductivities of GnP measured parallel and perpendicular to the surface are 10^7^ and 10^2^ S/m, respectively. *N*-methyl pyrrolidone (NMP), with a purity of 99%, a boiling point of 202 °C, and a flash point of 95 °C, was obtained from Panreac Química SA (Barcelona, Spain).

PEI-GnP foams were prepared containing 0.1–2.0 wt % GnP using scCO_2_ dissolution. To do so, prior preparation of a set of foam precursors with various GnP concentrations was carried out. The preparation of said foam precursors began with obtaining a GnP-rich PEI-GnP masterbatch. For that, a solution of NMP-GnP was ultrasonicated for 30 min using a FB-705 ultrasonic processor (Fisher Scientific, Hampton, NH, USA) at maximum amplitude with a 12 mm solid tip probe and 20 kHz operating frequency, and maintained at constant temperature of 50 °C using an ice-bath. PEI was added to the solution and dissolved at 75 °C while stirring using a magnetic stirrer at 450 rpm during 24 h. The resulting solution was filtered and rinsed with distilled water and, later, dried under vacuum at 140 °C (maximum vacuum drier temperature) for a week to remove any trace of the solvent. The final PEI-GnP masterbatch contained a GnP amount of 40 wt %.

PEI-GnP nanocomposites with variable concentrations of GnP (0.1–2.0 wt % GnP) were prepared by melt-mixing pure PEI with the PEI-GnP masterbatch using a Brabender Plastic-Corder (Brabender GmbH and Co., Duisburg, Germany). The procedure consisted in feeding 48 g of previously physically-mixed pure PEI and PEI-GnP masterbatch to the Brabender mixing chamber and initially melt-mixing for 6 min at 250 °C using a constant rotation speed of 30 rpm. Mixing continued for another 6 min at the same conditions in order to guarantee homogeneity of the mix, monitoring the temperature and torque values to confirm the stability of the process and the absence of possible degradation. Nanocomposites were then extracted from the mixing chamber and compression-molded into circular-shaped discs (foam precursors) with a nominal thickness of 3 mm and a diameter of 74 mm using a hot-plate press (PL15, IQAP LAP, IQAP Masterbatch Group S.L., Barcelona, Spain) at 300 °C and 70 bar during 4–5 min. 

**Figure 1:**
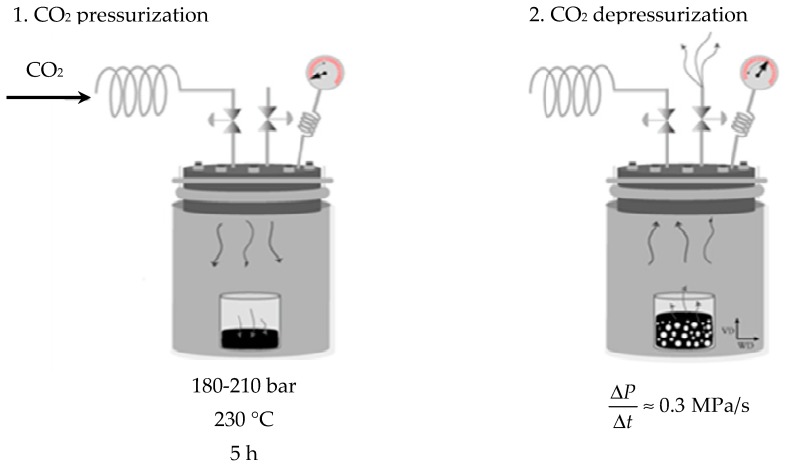
Scheme of the one-step scCO_2_ foaming process.

Foaming was carried out by placing the foam precursors inside a high pressure vessel (CH-8610 Uster/Schweiz, Büchiglasuster, Switzerland) using a one-step scCO_2_ dissolution process. Firstly, scCO_2_ dissolution was achieved by simultaneously raising the temperature and pressure of the vessel to 230 °C and 180–210 bar, respectively, and maintaining the temperature and pressure conditions for 5 h. Foaming took place by applying a sudden depressurization at a rate around 0.3 MPa/s and moderate controlled cooling of the vessel using a water cooling system. Figure 1 shows both steps of CO_2_ pressurization/heating and CO_2_ depressurization/cooling used in order to obtain PEI-GnP foams. Thin skin layers formed on both top and bottom of the resulting foams were carefully removed before characterization.

Samples coded as PEI correspond to pure PEI foams and the ones coded as GnP to PEI-GnP nanocomposite foams. In the case of the second, the number placed before GnP represents the amount of GnP in weight percentage; for instance, 0.1 GnP corresponds to PEI-GnP foam containing 0.1 wt % GnP.

The foam’s density values were measured using the ISO-845 standard procedure. The porosity values were directly calculated from the density values of the foam and respective unfoamed material according to the following expression:

(1) $$\mathrm{Porosity}\;(\%)=\left(1-\frac{\rho }{\rho_{s}}\right)\times 100$$

where *ρ* and *ρ*_s_ are the density of the foam and density of the respective unfoamed material, respectively (*ρ*/*ρ*_s_ is the so-called relative density). The cellular structure of the foams was analysed using a JEOL (Tokyo, Japan) JSM-5610 scanning electron microscope (SEM) applying a voltage of 10 kV and a working distance of 40 mm. Samples were brittle-fractured using liquid nitrogen and later coated with a thin layer of gold by sputter deposition using a BAL-TEC (Los Angeles, CA, USA) SCD005 sputter coater under an argon atmosphere. The values of the average cell size (*Φ*) were measured using the intercept counting method, explained in detail in [25]. Five ×300 magnification SEM micrographs were used for each sample. Cell nucleation density (*N*_0_, in cells/cm^3^) was calculated assuming an isotropic distribution of spherical cells according to:

(2) $$N_{0}={\left(\frac{n}{A}\right)}^{3/2}(\frac{\rho_{s}}{\rho })$$

where *n* is the number of cells counted in each SEM micrograph and *A* is the area of the SEM image in cm^2^. 

Wide-angle X-ray diffraction was used to evaluate the characteristic (002) diffraction plane of GnP and the possible crystallinity of PEI by a PANalytical diffractometer (Almelo, The Netherlands) running with CuKα (λ = 0.154 nm) at 40 kV and 40 mA. The scanning range was from 2° to 60° using a scan step of 0.033°.

The study of the thermal stability of the foams was done using a TGA/DSC 1 Mettler Toledo (Columbus, OH, USA) STAR System analyser with samples of around 10.0 mg, applying a heating ramp from 30 to 1000 °C at 10 °C/min under a nitrogen atmosphere (constant flow of 30 mL/min). The weight loss evolution with temperature was analysed using the STAR Evolution Software (Mettler Toledo Columbus, OH, USA). 

The study of the viscoelastic behaviour of the foams was performed using dynamic-mechanical-thermal analysis. Particularly, the foam’s storage and loss moduli (*E*′ and *E*″, respectively) were measured as a function of temperature, and PEI’s glass transition temperature (*T*_g_) was determined. A DMA Q800 from TA Instruments (New Castle, DE, USA) was used in a single cantilever configuration. Samples were analysed by heating at a rate of 2 °C/min from 30 to 300 °C while applying a dynamic strain of 0.02% and frequency of 1 Hz. Rectangular-shaped specimens used in this test had a length of 35.5 ± 1.0 mm, width of 12.5 ± 1.0 mm, and thickness of 3.0 ± 0.5 mm. Three different measurements were performed for each sample (error < 5%).

The electrical conductivity measurements were performed on 20 × 20 × 1 mm samples using a 4140B model HP pA meter/dc voltage source with a two-probe set. A thin layer of colloidal silver conductive paint was used to cover the surfaces of the samples in contact with the copper electrode pads, which had an electrical resistance between 0.01 and 0.1 Ω/cm^2^ to ensure perfect electrical contact. A direct current voltage was applied with a range of 0–20 V, voltage step of 0.05 V, hold time of 10 s and step delay time of 5 s. The electrical conductivity (*σ*, in S/m) was calculated using:

(3) $$\sigma =1/\rho_{v}$$

and

(4) $$\rho_{v}=RA_{E.C}/d$$

where *ρ*_v_ (Ω·m) is the electrical volume resistivity, *R* is the electrical resistance of the sample (in Ω), *A*_E.C_ is the area of the surface in contact with the electrode (in m^2^), and *d* is the distance between the electrodes (in m). A correction was applied to the measured values of electrical conductivity considering that porosity could affect the effective surface area in contact with the electrode. The average cell size and the cell density of foams were used in order to obtain the corrected value of electrical conductivity (*σ*_corr_) by taking into account variations in the effective surface area as follows:

(5) $$\sigma_{\mathrm{corr}}=\frac{d}{R(A_{non-cell}+A_{cell-hemisphere})}$$

where *A*_non-cell_ is the *A*_E.C_ with the cell section area excluded and 

(6) $$A_{cell-hemisphere}=\left(\frac{n}{A}\right)A_{E.C}\left(2\pi \frac{\Phi^{2}}{4}\right)$$

Therefore:

(7) $$A_{non-cell}+A_{cell-hemisphere}=A_{E.C}+(\left(\frac{n}{A}\right)A_{E.C}\left(\pi \frac{\Phi^{2}}{4}\right))$$

The values of *n*, *A*, and *Φ* were obtained from the previously analysed SEM micrographs, and represent the number of cells, the corresponding area of the micrograph, and the average cell size, respectively.

## 3. Results

### 3.1. Cellular Structure of the Foams

The composition of PEI-GnP nanocomposite foams, their respective relative densities and main cellular structure characteristics are presented in Table 1. 

Foams presented densities between 0.52 and 0.63 g/cm^3^ (relative densities between 0.40 and 0.49). Although the foam containing 2 wt % of GnP presented the highest relative density, no direct correlation was found between the relative density and the amount of GnP present in PEI-GnP foams. The porosity values were between 51.0% and 59.6%, with the minimum corresponding to 0.4 GnP and 2.0 GnP foams and the maximum to 1.5 GnP foam.

**Figure 2:**
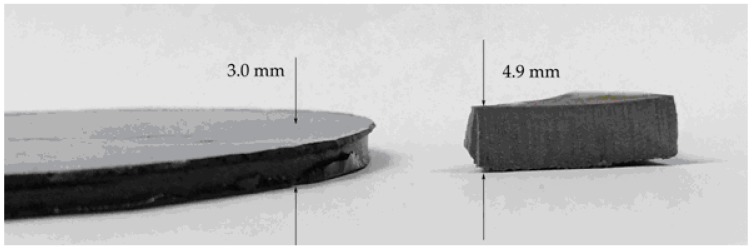
Digital photographs showing the sample before foaming (foam precursor, **left**) and after foaming (**right**).

**Figure 3:**
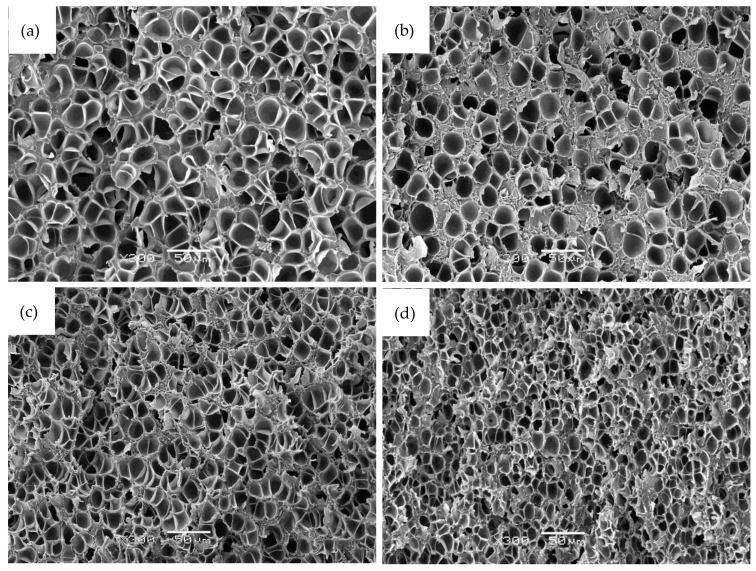
SEM micrographs at ×300 magnification illustrating the microcellular structure of (**a**) pure PEI; (**b**) 0.4 GnP; (**c**) 1.0 GnP; and (**d**) 2.0 GnP foams.

Digital photographs showing the sample before (foam precursor) and after foaming and characteristic SEM images showing the microcellular structure of PEI-GnP foams are respectively presented in Figure 2 and Figure 3. As can be seen, the addition of GnP seemed to induce the formation of cellular structures with slightly smaller cells.

The microcellular foams obtained in this process had an approximate average cell size around 10 µm, with the smallest cells corresponding to 0.7 GnP foam (5.4 µm) and the largest corresponding to pure PEI (14.0 µm). A slight reduction in the average cell size was observed between pure PEI and PEI-GnP foams, showing that the presence of GnP slightly affected the cellular structure of the resulting foams. Consequently, the highest cell nucleation density value corresponded to 0.7 GnP foam (7.0 × 10^9^ cells/cm^3^) and the lowest to PEI (3.9 × 10^8^ cells/cm^3^).

The peak intensity and full width at half maximum (FWHM) values of X-ray diffraction spectra related to the (002) characteristic diffraction plane of GnP found at 2θ = 26.5° are presented in Table 2. The low and stretched (002) peak formation shows that by applying ultrasonication, melt-mixing, and foaming through scCO_2_, a significant improvement in dispersion of GnP in foams containing up to 1.5 wt % of GnP could be achieved.

**Figure 4:**
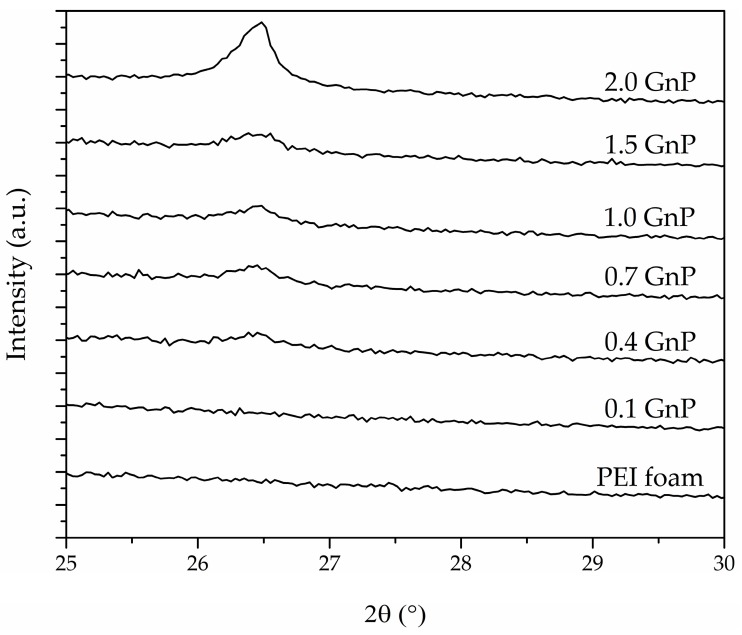
X-ray spectra of PEI and PEI-GnP nanocomposite foams illustrating the disappearance or reduction in intensity of GnP’s (002) characteristic diffraction plane.

Disappearance and/or significant reduction in intensity of the (002) characteristic diffraction plane of GnP for foams containing up to 1.5 wt % of GnP (see Figure 4) showed that the ultrasonication process was effective and provided a better dispersion and partial exfoliation of GnP stacks. Additionally, melt-mixing and sudden depressurization during foaming could have promoted further dispersion due to shear forces applied during these steps. Nevertheless, the GnP’s (002) diffraction plane peak was clearly visible in the 2.0 GnP foam, which was related to the not full dispersion of GnP nanoplatelets, as at such high GnP concentration the ultrasonication, melt-mixing and foaming stages were not enough to guarantee a proper dispersion of the nanoplatelets throughout the polymer matrix.

This high level of GnP dispersion was possible through the combination of ultrasonication, melt-mixing and one-step foaming of PEI-GnP nanocomposites. In the primary stages of foam precursor preparation, GnP stacks were forced to separate by applying ultrasonication. Afterwards, during melt-mixing, high shear forces were applied, favouring GnP distribution and dispersion throughout the polymer matrix. The ultimate stage of one-step scCO_2_ foaming could have further induced GnP dispersion and partial exfoliation.

### 3.2. Thermal Analysis

**Figure 5:**
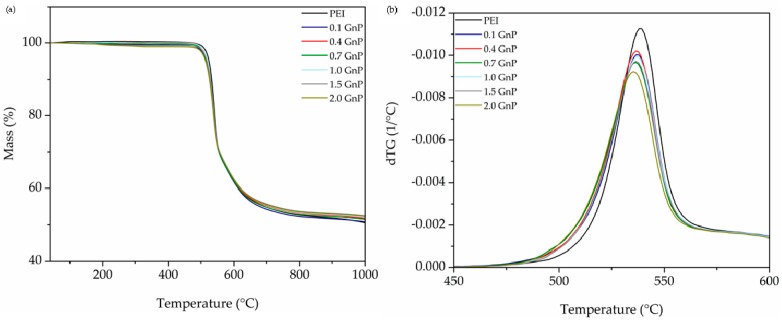
(**a**) TGA and (**b**) dTG thermograms of pure PEI and PEI-GnP nanocomposite foams.

Typical thermogravimetric curves (TGA) of decomposition of all foams are displayed with their respective first derivative (dTG) in Figure 5. The values of the temperature corresponding to a 5% weight loss (*T*_5% weight loss_), the temperature at maximum velocity of degradation (*T*_max_), the temperature corresponding to a 35% weight loss (*T*_35% weight loss_), the char residue (*CR*, in wt %), and the limiting oxygen index (LOI), calculated based on Van Krevelen and Hoftyzer [26] equation:

(8) LOI(%)=17.5+0.4CR

are presented in Table 3. Results indicate a characteristic two-step thermal degradation of PEI, with the first step being related to the decomposition of the aliphatic part of PEI followed by the degradation of the aromatic part in a second step [4,5]. 

As can be seen in Table 3 and Figure 5, there were minor changes in the degradation behaviour of foams regarding the addition of GnP. It has been reported that the addition of carbon-based nanoparticles, especially platelet-like, such as graphene nanoplatelets, could result in improved thermal stability of nanocomposites through a better physical barrier effect, hindering the escape of volatile gasses during pyrolysis [27]. On the other hand, the high thermal conductivity of GnP could promote a higher heat transfer velocity through the foamed structure, thus resulting in a faster degradation [6]. As can be seen, at the first stage of decomposition there was a slight general decreasing trend in *T*_5% weight loss_ and *T*_max_ with increasing GnP amount, suggesting an accelerating effect of GnP on the degradation behaviour; however, as the degradation process approached the second stage, a reversed relation was observed, resulting in a delay in the values of *T*_35% weight loss_ and a rise in *CR*. This behaviour is the result of the mentioned contradictory factors, one being the barrier effect of GnP and the other the enhanced heat transfer, which simultaneously affect the thermal degradation, their importance varying in the different stages of decomposition. This could be verified by the calculated values of LOI presented in Table 3, which showed an increasing trend with incrementing the amount of GnP.

### 3.3. Dynamic-Mechanical-Thermal Behavior

The results of the dynamic-mechanical-thermal analysis (DMTA) of all foams are presented in Table 4. For comparative purposes, the DMTA results of PEI-based nanocomposite foams previously prepared by the WVIPS method are also presented [5,6]. As with PEI-GnP foams prepared by one-step scCO_2_ foaming, the prefix number represents the amount of nanoparticle in wt %, followed by the type of nanoparticle (GnP or CNT) and the letters S and NS, representing whether ultrasonication was applied or not, respectively. 

The results indicate that two main factors could have affected the viscoelastic response of foams: Their relative density and the amount of GnP. The foam’s glass transition temperature (*T*_g_) was obtained from the temperatures corresponding to the maximum of the loss modulus (Max *E*″) and tanδ (Max tanδ) curves. The storage modulus (*E*′) was obtained from the DMTA curves at 30 °C. The specific storage modulus values (*E*′_spec_) of foams were calculated by dividing *E*′ obtained at 30 °C by their respective density.

**Figure 6:**
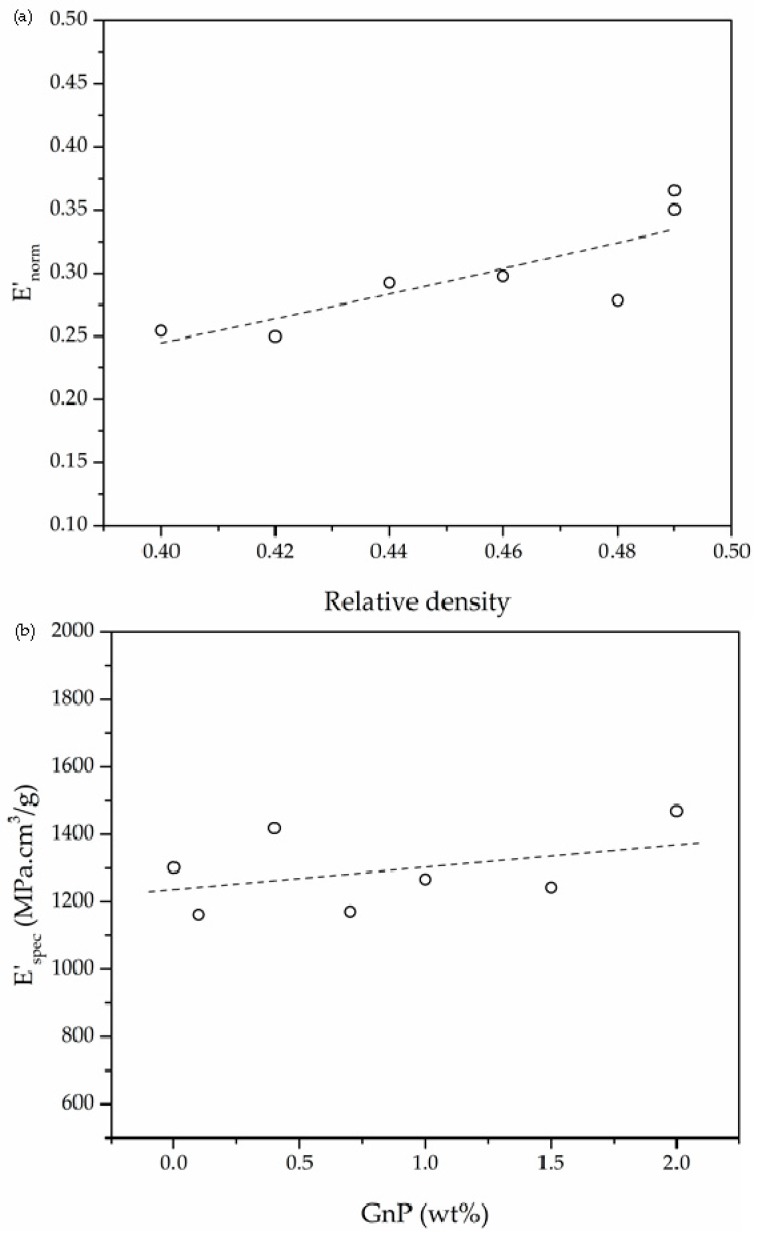
(**a**) Effect of relative density on the normalized storage modulus of PEI and PEI-GnP nanocomposite foams and (**b**) enhancement of the specific modulus with increasing GnP content.

As can be seen in Figure 6a, foams showed and increasing trend of the normalized modulus (*E*′_norm_), defined as the quotient between the storage modulus of the foam and the storage modulus of the respective unfoamed material (*E*′_s_), i.e., *E*′_norm_=*E*′/*E*′_s_, with increasing relative density. Additionally, Figure 6b illustrates the effect of GnP weight percentage on the specific modulus of the foams, where a general increasing trend was observed with incrementing the amount of GnP.

Regarding the viscous part, on the one hand the temperature corresponding to the maximum value of the loss modulus did not change significantly with increasing the amount of GnP. On the other hand, a general increasing trend was observed for the temperature corresponding to the maximum of tanδ with incrementing GnP’s concentration, rising 5 °C for the 2.0 GnP foam compared to that of pure PEI foam. Graphene has proven capable of hindering the molecular mobility in multiple study cases of nanocomposites [28,29,30] due to possible strong interfacial interaction and/or chemical bonding between graphene and the polymeric matrix. Hence, the increase in the temperature corresponding to the maximum of tanδ could be related to an improved interaction of the polymer with graphene nanoplatelets, broadening the glass transition temperature window.

It is commonly known that density, and hence relative density, plays a key role in the mechanical performance of foams. Here, as can be seen in Table 4, relative density appears to have a clear impact on the storage modulus of foams. For instance, the storage modulus increased significantly from 630.7 MPa to 922.1 MPa with increasing relative density from 0.42 to 0.49. Gibson and Ashby [31] introduced a model that represents the relation of the elastic modulus with density for closed-cell foams assuming foams as a cubic array of individual units:

(9) $$\frac{E}{E_{s}}\approx C{\left(\frac{\rho }{\rho_{s}}\right)}^{n}$$

where *ρ* and *E* are respectively the density and the elastic modulus of the foams and *ρ*_s_ and *E*_s_ correspond to those of the respective unfoamed material. In this equation *C* represents the geometry constant of proportionality and is commonly assumed to be equal to 1 [31].

As presented in Figure 6a, with a power fit of the normalized storage modulus (*E*′_norm_) obtained at 30 °C as a function of relative density, the value of exponent *n* could be calculated. This value is related to the efficiency of foaming, with values around 1 representing a smoother decrease in the normalized elastic modulus with reducing density, typical of homogenous closed-cell foams with relatively small cell sizes [32]. Values close to 2 represent a faster decrease of *E*′_norm_ with reducing density. The *n* value in this series of foams was equal to 1.53, somewhat close to homogeneous closed-cell structure with few interconnectivities and irregularities. However, this model does not take into account the eventual effects of an additional component, in this case GnP, on the mechanical properties of the foams, nor its secondary effects on the cellular morphology of the foams. Therefore, in order to address the effects of GnP on the mechanical performance of the foams, the specific storage modulus (*E*′_spec_) was calculated and represented as a function of GnP content (Figure 6b). As expected based on the inherently high elastic modulus of GnP, a general increasing tendency was observed with the addition of GnP.

Interestingly, PEI-GnP foams containing 1.0 and 2.0 wt % GnP showed higher values of *E*′_spec_ when compared to their counterparts prepared using WVIPS method containing the same amount of GnP [6]. Additionally, PEI-GnP foams presented much higher values of both *E*′ and *E*′_spec_ for similar relative densities at lower GnP amounts when compared to foams containing 2.0 wt % of CNT prepared by the WVIPS method [5] (in both cases, compare values presented in Table 4). This could suggest that a more effective reinforcing effect could be achieved by adding GnP when compared to CNT and guaranteeing a more homogeneous microcellular structure via scCO_2_ foaming when compared to WVIPS method.

### 3.4. Electrical Conductivity

It has been suggested that mainly two factors affect the electrical conductivity of polymer-based foams containing conductive carbon-based nanoparticles: Firstly and most importantly, the dispersion level of the conductive nanoparticles; and secondly, the porosity level of the foams.

**Figure 7:**
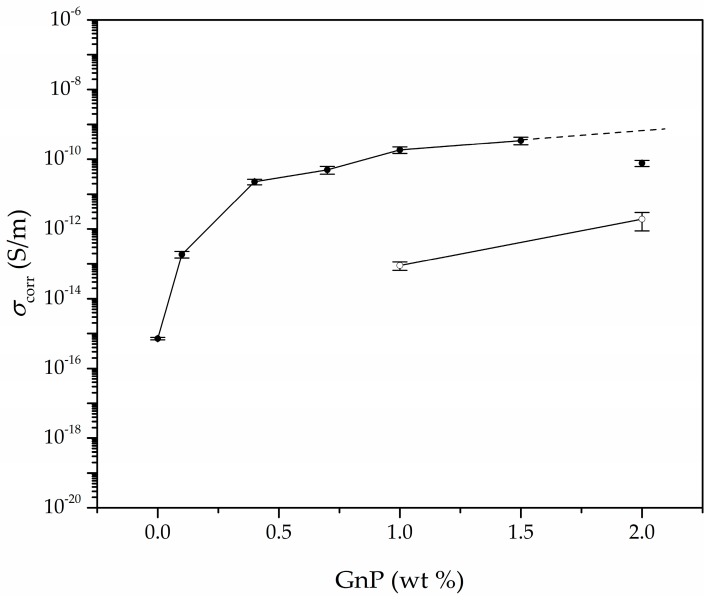
Electrical conductivity enhancement of PEI and PEI-GnP nanocomposite foams with increasing GnP amount. Black circles represent the electrical conductivity values corresponding to foams prepared by one-step scCO_2_ foaming and white circles correspond to the electrical conductivity of foams prepared by WVIPS [6].

As can be seen in Table 5 and Figure 7, the addition of increasingly higher GnP amounts up until 1.5 wt % GnP led to foams with increasingly higher electrical conductivities, related to the formation of a more effective conductive network attained by the higher amount of GnP and proper GnP dispersion throughout the cell walls after foaming. Nevertheless, comparatively 2.0 GnP foam displayed a lower conductivity than 1.0 GnP or 1.5 GnP foams, which was related to a certain GnP aggregation at the highest added GnP concentration (2.0 wt % GnP) after foaming, as previously demonstrated by the intense (002) GnP crystal diffraction plane in 2.0 GnP foam (see Figure 4).

As mentioned before, ultrasonication was used in the preparation of the PEI-GnP masterbatch in order to promote a high level of GnP dispersion. This method has proven worthy in enhancing the electrical conductivity of foams by reducing particle agglomeration [6,33,34,35,36]. Ultrasonication, combined with melt-mixing and sudden pressure drop applied during foaming were responsible for promoting the dispersion and partial exfoliation of GnP in PEI, as previously experienced in foams prepared via scCO_2_ foaming [21]. 

**Figure 8:**
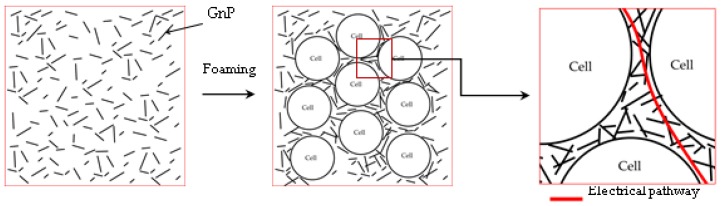
Detail of GnP dispersion showing the microstructural changes in a polymer foam containing GnP with foaming and the effects in electrical conduction.

Moreover, the porosity of foams could play a key role in the inter-particle distance between GnP nanoparticles, enhancing their electrical conductivity by forming a more compact network of conductive nanoparticles. In this sense, by increasing the porosity GnP nanoparticles present in the continuous polymer phase of the foam would be pushed closer together, favouring the formation of an electrical pathway, as shown in Figure 8. 

As can be seen, the electrical conductivity of the foams increased by six orders of magnitude, reaching 3.45 × 10^−10^ S/m with the addition of only 1.5 wt % (0.35 vol %) GnP. Interestingly, this value was clearly higher than those obtained for foams containing the same amount of GnP prepared by WVIPS method [6]. Since ultrasonication was used in both cases, this result could indicate an enhanced dispersion of GnP nanoparticles via melt-mixing and formation of a more effective conductive network throughout the foam using one-step scCO_2_ foaming. 

In terms of electrical conductivity models, a tunnelling mechanism seemed more accurate compared to a percolative model. Although the percolative model has been used vastly to explain the conductivity behaviour in various studies of nanocomposites containing carbon nanotubes [5,37,38,39] and graphene [40,41,42,43], this model is applicable only when the concentration of the conductive filler is above the critical volume fraction (*ϕ_c_*), also known as the percolation threshold. The percolative model is based on the physical contact between conductive nanoparticles in order to form a pathway for the electrical conduction and is expressed as:

(10) $$\sigma \propto (\phi -\phi_{c}{)}^{\nu }$$

where the electrical conductivity value (*σ*) is proportional to the volume fraction of the conductive filler (*ϕ*) and the percolation threshold (*ϕ_c_*), and *ν* is the percolation exponent [43]. Nevertheless, a tunnel conduction model was preferred, as it has been proven to be a more accurate model to anticipate the electrical conductivity behaviour of nanocomposite foams containing conductive carbon-based nanoparticles. As mentioned in some of our previous works [4,6], this model was assumed as the main conduction mechanism in this series of foams due to two main reasons: Firstly, GnP’s concentration was below the percolation threshold, resulting in absolute electrical conductivities clearly below what would be expected assuming physical contact between conductive nanoparticles. Secondly, the percolation model does not consider that these nanocomposite foams have already achieved a certain level of electrical conductivity for GnP concentrations below the critical value.

According to quantum mechanics, a tunnelling mechanism indicates that when there is an absence of physical contact between conductive particles the electrons still have the possibility to penetrate through a potential barrier. The crossing of electrons in a tunnelling model could occur when the applied electric field possesses enough potential so that the electron wave function could penetrate the barrier [44]. Assuming a tunnel-like mechanism, the dc electrical conductivity can be expressed as [45,46,47]:

(11) σ∝exp(−Ad)

where *A* and *d* are the tunnel parameter and tunnel distance, respectively. 

**Figure 9:**
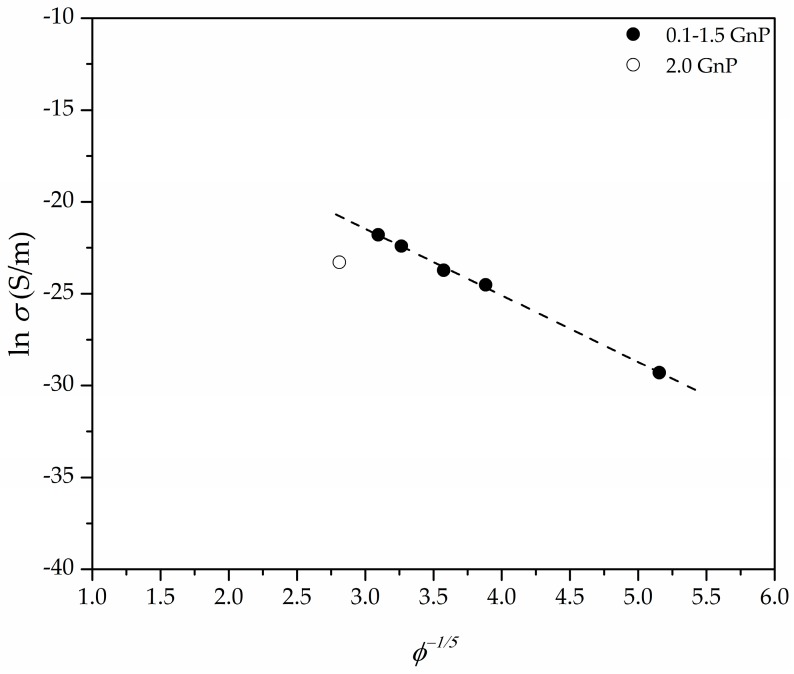
Representation of the fitting results of the electrical conductivity versus *ϕ*^−1/5^, demonstrating the tunnel conduction characteristic of a 3D random particle distribution system formed by conductive GnP nanoparticles.

The *ϕ*^−*n*^ presented in Figure 9 (assuming *n* = 1/5) is directly proportional to the tunnel distance (*d*), where the value of *n* is related to the geometry of the conductive fillers and their distribution. Particularly, the value of *n* for randomly distributed spherical-shaped particles has been proposed to be equal to 1/3 [48], while the value of *n* = 1 corresponds to a 3D random fibre network [49]. We have shown in our previous works [4,6] that assuming a tunnel-like approach for PEI-GnP foams prepared using WVIPS led to a value of *n* equal to 1/5, which, according to Krenchel [50] and Fisher et al. [51], could confirm the existence of a conductive network formed by GnP with a 3D random distribution. Similarly, in this work the best fit was obtained using an *n* value of 1/5 (see fitting representation in Figure 9). As shown in previous works, the combination of ultrasonication and increased porosity due to foaming promoted GnP dispersion and led to enhanced electrical conductivity values. In this work it was observed that using one-step scCO_2_ foaming the electrical conductivity could be improved by a few orders of magnitude for low GnP amounts (<2 wt %), explained on the basis of the already mentioned improved dispersion of GnP.

## 4. Conclusions

In terms of cellular structure, PEI-GnP foams presented a microcellular closed-cell structure with similar cell sizes and cell densities independently of the amount of GnP.

The X-ray diffraction analysis showed that the combination of ultrasonication, melt-mixing, and sudden expansion using depressurization of scCO_2_ promoted enhanced dispersion and partial exfoliation of GnP in the foams.

The addition of GnP has shown to have opposed influences on the thermal degradation behaviour of the foams. On the one hand, the char residue resulting from burning increased with incrementing the amount of GnP while, on the other hand, the value of *T*_max_ experienced a minor decrease with augmenting GnP content. These behaviours could relate to both the barrier effect of platelet-like GnP, hindering the escape of volatile gases, and the increase in heat transfer due to the presence of the highly conductive GnP, resulting in a faster degradation.

The dynamic-mechanical-thermal performance of PEI-GnP foams was globally controlled by their relative density, as they displayed increasingly higher specific storage moduli with increasing relative density independently of GnP’s content. Only for similar relative densities the addition of higher GnP amounts led to stiffer foams. Comparatively, PEI-GnP foams prepared by scCO_2_ dissolution presented much higher storage moduli at lower GnP concentrations than foams containing 2 wt % CNT prepared by WVIPS method [5], explained mainly by their more homogeneous microcellular structure.

The electrical conductivity of foams increased significantly with incrementing GnP’s content, following a tunnel-like conduction mechanism. Foams showed greater values when compared to foams previously prepared using WVIPS method. This increase is believed to be a consequence of the enhanced dispersion of GnP by the combination of ultrasonication, melt-mixing and sudden pressure drop applied during one-step scCO_2_ foaming, confirmed by X-ray diffraction results. One-step scCO_2_ foaming could have also promoted increases in the electrical conductivity by decreasing the effective distance between conductive nanoparticles for electrical conduction, as the growth of cells pushed GnP nanoparticles closer to each other within the continuous PEI matrix.

The foams prepared and analysed in this work could be used in cutting-edge sectors, such as aerospace or telecommunications, for applications involving ESD or EMI shielding due to their combination of medium-low density and enhanced electrical conductivity.

## Figures and Tables

**Table 1:** Composition, relative densities, and cellular structure characteristics of PEI and PEI-GnP nanocomposite foams.

Sample	GnP (wt %)	GnP (vol%)	Relative Density	*Φ* (μm) ^1^	*N_0_* (cells/cm^3^)
PEI	0.0	0.00	0.44	14.0 (5.0)	5.1 × 10^8^
0.1 GnP	0.1	0.03	0.48	11.7 (4.2)	5.6 × 10^8^
0.4 GnP	0.4	0.11	0.49	13.6 (4.4)	3.9 × 10^8^
0.7 GnP	0.7	0.17	0.42	5.4 (2.3)	6.5 × 10^9^
1.0 GnP	1.0	0.27	0.46	9.5 (3.3)	1.1 × 10^9^
1.5 GnP	1.5	0.35	0.40	10.0 (4.0)	1.2 × 10^9^
2.0 GnP	2.0	0.57	0.49	7.5 (2.9)	1.6 × 10^9^

^1^ Standard deviation of the average cell size is presented between parentheses.

**Table 2:** Intensity and FWHM values of the characteristic (002) crystal diffraction plane of GnP in PEI and PEI-GnP nanocomposite foams.

Sample	Intensity (a.u.)	FWHM (°)
PEI	-	-
0.1 GnP	-	-
0.4 GnP	350.5	0.23
0.7 GnP	481.2	0.35
1.0 GnP	505.4	0.30
1.5 GnP	483.5	0.40
2.0 GnP	1716.6	0.29

**Table 3:** TGA results of pure PEI and PEI-GnP nanocomposite foams.

Sample	Decomposition Temperature (°C)			*CR* (wt %)	LOI (%)
	*T*_5% weight loss_	*T*_max_	*T*_35% weight loss_		
PEI	523.4	538.7	580.9	50.5	37.7
0.1 GnP	516.8	537.3	578.3	50.8	37.8
0.4 GnP	518.7	536.9	580.4	51.7	38.2
0.7 GnP	516.2	536.5	579.3	51.5	38.1
1.0 GnP	519.0	537.0	583.0	51.9	38.3
1.5 GnP	517.4	536.6	582.9	52.4	38.5
2.0 GnP	513.9	535.3	581.7	52.3	38.4

**Table 4:** DMTA results of pure PEI and PEI-GnP nanocomposite foams.

Sample	Relative Density	*E*′ at 30 °C (MPa)	*E*′_spec_ (MPa·cm^3^/g)	Glass Transition (°C)	
				Max *E*″	Max tanδ
scCO_2_ foams					
PEI	0.44	738.6	1295.8	212.0	220.9
0.1 GnP	0.48	702.8	1171.3	212.1	224.4
0.4 GnP	0.49	884.3	1426.3	212.5	221.4
0.7 GnP	0.42	630.7	1168.0	212.6	224.0
1.0 GnP	0.46	751.6	1273.9	212.8	218.2
1.5 GnP	0.40	642.3	1235.2	213.0	224.2
2.0 GnP	0.49	922.1	1463.7	210.9	226.5
WVIPS foams ^1^					
1.0 GnP NS	0.44	742.6	1335.6	218.0	225.0
2.0 GnP NS	0.39	568.1	1147.7	218.4	226.7
1.0 GnP S	0.26	370.4	1110.9	223.1	229.8
2.0 GnP S	0.26	385.3	1170.5	223.3	228.6
2.0 CNT S	0.44	442.9	776.5	221.5	227.1

^1^ PEI-based nanocomposite foams prepared by water vapour induced phase separation (WVIPS) [5,6].

**Table 5:** Electrical conductivity and corrected electrical conductivity values of pure PEI and PEI-GnP nanocomposite foams.

Sample	GnP (wt %)	GnP (vol%)	Porosity (%)	*σ* (S/m)	*σ*_corr_ (S/m) ^1^
PEI	0.0	0.00	55.3	7.18 × 10^−16^	4.60 × 10^−16^ (9.92 × 10^−17^)
0.1 GnP	0.1	0.03	52.4	2.70 × 10^−13^	1.88 × 10^−13^ (4.03 × 10^−14^)
0.4 GnP	0.4	0.11	51.0	3.17 × 10^−11^	2.27 × 10^−11^ (4.10 × 10^−12^)
0.7 GnP	0.7	0.17	57.7	7.16 × 10^−11^	4.99 × 10^−11^ (1.25 × 10^−11^)
1.0 GnP	1.0	0.27	53.5	3.76 × 10^−10^	1.86 × 10^−10^ (4.00 × 10^−11^)
1.5 GnP	1.5	0.35	59.6	5.12 × 10^−10^	3.45 × 10^−10^ (8.67 × 10^−11^)
2.0 GnP	2.0	0.57	51.0	1.12 × 10^−10^	7.70 × 10^−11^ (1.53 × 10^−11^)

*σ*_corr_ represents the electrical conductivity corrected according to Equation (7). **^1^** Standard deviation of the corrected electrical conductivity is presented between parentheses.
